# Cool and Hot Executive Function Impairments in Violent Offenders with Antisocial Personality Disorder with and without Psychopathy

**DOI:** 10.1371/journal.pone.0065566

**Published:** 2013-06-20

**Authors:** Stephane A. De Brito, Essi Viding, Veena Kumari, Nigel Blackwood, Sheilagh Hodgins

**Affiliations:** 1 School of Psychology, University of Birmingham, Birmingham, United Kingdom; 2 Division of Psychology and Language Sciences, University College London, London, United Kingdom; 3 Department of Psychology, Institute of Psychiatry, King's College London, London, United Kingdom; 4 Department of Forensic and Neurodevelopmental Science, Institute of Psychiatry, King's College London, London, United Kingdom; 5 Département de Psychiatrie, Université de Montréal, Montréal, Québec, Canada; Bellvitge Biomedical Research Institute-IDIBELL, Spain

## Abstract

**Background:**

Impairments in executive function characterize offenders with antisocial personality disorder (ASPD) and offenders with psychopathy. However, the extent to which those impairments are associated with ASPD, psychopathy, or both is unknown.

**Methods:**

The present study examined 17 violent offenders with ASPD and psychopathy (ASPD+P), 28 violent offenders with ASPD without psychopathy (ASPD−P), and 21 healthy non-offenders on tasks assessing cool (verbal working memory and alteration of motor responses to spatial locations) and hot (reversal learning, decision-making under risk, and stimulus-reinforcement-based decision-making) executive function.

**Results:**

In comparison to healthy non-offenders, violent offenders with ASPD+P and those with ASPD−P showed similar impairments in verbal working memory and adaptive decision-making. They failed to learn from punishment cues, to change their behaviour in the face of changing contingencies, and made poorer quality decisions despite longer periods of deliberation. Intriguingly, the two groups of offenders did not differ significantly from the non-offenders in terms of their alteration of motor responses to spatial locations and their levels of risk-taking, indicated by betting, and impulsivity, measured as delay aversion. The performance of the two groups of offenders on the measures of cool and hot executive function did not differ, indicating shared deficits.

**Conclusions:**

These documented impairments may help to explain the persistence of antisocial behaviours despite the known risks of the negative consequences of such behaviours.

## Introduction

Most violent crimes are committed by a small group of males who display persistent antisocial and aggressive behaviour from childhood onwards [1], [2]. This life-long pattern of behaviour is indexed by DSM-IV diagnoses of Conduct Disorder (CD) prior to age 15 and Antisocial Personality Disorder (ASPD) in adulthood [3]. Life-long patterns of risk taking and impulsivity are central features of ASPD [4]. Illegal behaviours persist despite repeated criminal sanctions. Neuropsychological deficits in executive function (EF) reflecting the higher order cognitive control of thought, action, and emotion [5] have been hypothesized to be central to the onset and persistence of severe antisocial and aggressive behaviour [6]–[11].

There is accumulating evidence that men with ASPD represent a heterogeneous population with respect to personality traits, aggressive behaviour, offending patterns, [4], and engagement with, and response to, cognitive-behavioural offender rehabilitation programs [12], [13]. While all within this population present an early onset of antisocial behaviour that remains stable over the life-span, a sub-group additionally present psychopathy (ASPD+P), as defined by the Psychopathy Checklist-Revised (PCL-R; [14], [15]). Psychopathy is a syndrome characterized by a constellation of affective, interpersonal, and behavioural features [14], including a lack of empathy, callousness, shallow affect and a failure to take responsibility for one's actions, and a pathological interpersonal style involving grandiosity, glibness, superficial charm, and the manipulation of others [16]. Much research has demonstrated that in comparison to offenders without psychopathy, those with ASPD+P begin offending at a younger age [17], more often engage in instrumental aggression [18], and acquire more convictions or charges for violent offences [19], [20].

Consistent with the differences in personality traits and aggressive behaviour that distinguish adult men with ASPD+P and those with ASPD and not psychopathy (ASPD−P), recent evidence suggests that the two groups show distinct emotional impairments [20], affective processing [21], brain response to emotional stimuli when engaged in goal-directed behaviour [22], and structural brain anomalies [23]. Those with ASPD−P are hypothesized to present a low threshold for engaging in reactive aggressive behaviour towards others due to a hyper-sensitivity to threat, both real and perceived, as evidenced by hyperactivity in the amygdala [6]. By contrast, individuals with ASPD+P show hypo-activity in the amygdala in response to threat (e.g., [24]). Importantly, these distinct phenotypes emerge early in childhood [25]. The lack of responsiveness in the amgydala, especially to fearful faces, among adults with ASPD+P (e.g., [26]) and children showing the antecedents of this condition (e.g., [27], [28]) may underlie their impairment in stimulus-reinforcement learning central to passive avoidance paradigms [29]. This impairment has been hypothesized to be a core deficit of ASPD+P that emerges in childhood and limits learning not to engage in instrumental antisocial and aggressive behaviour and learning to engage in prosocial behaviour [29]. Evidence from developmental studies examining children and adolescents is indeed consistent with the notion that different EF impairments are associated with distinct forms of antisocial behaviour and patterns of aggressive behaviour [30], [31].

Cool EF refers to top-down processes subsumed primarily by the dorsolateral prefrontal cortex (DLPFC) and ventrolateral PFC that are distinctly cognitive in nature and usually elicited by abstract, decontextualized problems. Working memory, response inhibition, planning, sustained attention, and attentional set-shifting are considered to be cool EF [32], [33]. By contrast, hot EF refers to cognitive processes that have an affective, motivational, or incentive/reward component; these processes are generally considered to be subsumed by ventromedial pathways connecting mesolimbic reward circuitry, including the amygdala and striatum, to the ventromedial prefrontal cortex (VMPFC) [34]. Appraising the motivational significance of a stimulus in affective decision-making paradigms and reappraising it in response reversal paradigms are considered hot EF [32], [35].

The extent to which cool and hot EF differ in the two types of violent offenders is difficult to determine from the extant literature as few studies have directly compared EF of violent offenders with ASPD−P and ASPD+P. Most previous studies compared offenders with ASPD+P to offenders without psychopathy([36], [37], but see [38]), while others compared individuals with ASPD from the community who had not been assessed for psychopathy (but see [39], [40] for two studies on violent offenders) to either healthy non-offenders [41]–[44] or patients without ASPD with substance use disorders [45], [46].

A large number of studies suggest that offenders with ASPD+P, in comparison to offenders without psychopathy, do not present impairments in cool EF such as attentional set-shifting, planning, and verbal working memory indexing the functional integrity of the DLPFC [47]–[53]. These studies, however, show that offenders with ASPD+P present impairments in cool EF such as response inhibition (e.g., [50]) and in hot EF tasks such as response reversal, behavioural extinction, and affective decision-making indexing the functional integrity of the VMPFC (e.g., [51], [52], [54], [55]). Further evidence of hot EF impairments among offenders with ASPD+P comes from studies showing that they make more commission errors (i.e., responses to stimuli paired with negative reinforcement) than non-psychopathic offenders on passive avoidance learning tasks assessing stimulus-reinforcement-based decision-making.

Only two studies have been published that examined violent offenders with clearly delineated ASPD−P [39], [40]. The majority of investigations of cool EF suggest that men with ASPD−P perform like healthy men on attentional set-shifting tasks, planning and measures of verbal or spatial working memory, all dependent on DLPFC functioning ([39], [41], [45], [56]; but see [57], [58]). By contrast, there is consistent evidence that men with ASPD−P are characterized by hot EF deficits as indicated by their impaired performance on tasks such response reversal and affective decision-making indexing the functional integrity of the VMPFC [43], [44], [46], [57].

To date, only one study [40] has compared violent offenders with ASPD+P and violent offenders with ASPD−P to healthy participants on tasks assessing cool EF (the Stockings of Cambridge planning task, attentional set-shifting on the intra-dimensional/extradimensional [ID/ED] set-shifting task, behavioural inhibition on a Go/No-Go task) and hot EF (response reversal components of the ID/ED task). Based on their scores on the Psychopathy Checklist: Screening Version (PCL: SV; [59]) the violent offenders with ASPD were divided into three groups (‘low’  =  PCL: SV ≤15, ‘medium’  =  PCL: SV  = 16–19; and ‘high’  =  PCL: SV >19). Results indicated that, regardless of psychopathy scores, offenders with ASPD, as compared to the healthy participants, exhibited subtle impairments in cool EF (planning, attentional set-shifting, response inhibition), but no hot EF impairment (i.e., reversal learning). In correlational analyses psychopathy scores were not related to performance on any of the tasks. Taken together, the results of this investigation suggested that violent offenders with ASPD+P and those with ASPD−P exhibit similar cool EF impairments as measured by attentional set-shifting, and similar performance on one index of hot EF (reversal learning). However, in view of the results Dolan [40] concluded that “further studies using a range of DLPFC and VMPFC tasks” (p.8) were needed.

Knowledge of cool and hot EF that are impaired or preserved in each type of violent offender could be used to improve the effectiveness of rehabilitation programs aimed at reducing recidivism [60]. While cognitive-behavioural programs have been shown to reduce criminal recidivism [61], [62], offenders with ASPD+P fail to benefit [12], [13]. Further, such knowledge will contribute to unravelling the etiology of persistent violent behaviour that is a prerequisite for preventing it.

The present study employed a broad range of neuropsychological tests to assess both cool and hot EF among violent offenders with ASPD+P, violent offenders with ASPD−P, and healthy non-offenders. Tests of EF were selected because they have been validated in studies of subjects with lesions in specific brain regions, all but one (Cambridge Gamble Task [CGT]) have been used in previous studies of ASPD+P or ASPD−P, they index processes that have been shown to play an important role in the display of aggressive behaviour (e.g., impairment in working memory), or to be related to core features of either ASPD+P or ASPD−P (e.g., insensitivity to punishment, impulsivity and risk-taking) (Detailed justifications available online in Text S1).

Both violent offenders with ASPD+P and ASPD−P exhibit life-long antisocial behaviour, but they are characterized by differences in personality traits, aggressive behaviour, emotion processing, and in response to interventions aimed at reducing antisocial/criminal behaviour. Consequently, we reasoned that they would show both similarities and differences in neurocognitive performance. First, we hypothesized that both the violent offenders with ASPD+P and those with ASPD−P would show similarly poor performance on two tests assessing cool EF (the Digit Span – Backward and Spatial Alternation Task), and on several tests of hot EF, (CGT, more reversal errors on the Probabilistic Response Reversal Task, more commission on the Passive Avoidance Learning Task) as compared to healthy non-offenders. Second, we hypothesized that the two groups of violent offenders would show one important difference in performance on these tests of hot EF. Consistent with much previous evidence and theorizing about psychopathy [6], [37], we hypothesized that the violent offenders with ASPD+P would make more commission errors on the Passive Avoidance Task than the violent offenders with ASPD−P.

## Materials and Methods

### Participants

Violent male offenders with ASPD and healthy male non-offenders with English as a first language were recruited from the community for a study of the neurobiological correlates of persistent aggression. Diagnostic interviews indicated that none had a life-time history of severe mental illness or a substance use disorder in the past month, and showed that all obtained a score of 70 or higher on the Wechsler Adult Intelligence Scale (WAIS-III; [63]).

#### Violent offenders with ASPD

Violent offenders were recruited from the National Probation Service. Probation officers identified potential participants with convictions for violent offences (murder, rape, attempted murder, and grievous bodily harm) confirmed by official criminal records. Offenders with a diagnosis of ASPD who obtained a total PCL-R score ≥25 were assigned to the ASPD+P group (n = 17), and those with a score ≥25 were assigned to the ASPD−P group (n = 28).

#### Healthy non-offenders

Non-offenders (n = 21) were recruited by means of advertisements in local newspapers and notices in the community. Those retained for the study had no criminal record, no mental disorder other than past substance misuse, and a PCL-R score of 24 or less.

### Classification measures

#### Structured clinical interview for DSM-IV

All participants completed the Structural Clinical Interview for DSM-IV, I and II, (SCID; [64]) administered by trained forensic psychiatrists to provide life-time and current DSM-IV diagnoses.

#### Psychopathy checklist – revised

The PCL-R [16] consists of 20 items that are scored by a trained rater on the basis of a file review and a semi-structured interview. Each of the 20 items is scored on a three-point scale (0–2), with the total score ranging from 0 to 40. Consistent with a validation study [65], a score of 25 or higher identified the syndrome of psychopathy among these European offenders. Forensic psychiatrists and psychologists trained to use the PCL-R administered interviews and extracted information from files in order to rate the scale. Interviews were videotaped and a random 25% sample was rerated by a second trained psychologist. Intra-class correlation coefficient values for PCL-R total scores were acceptable (0.81). Scores for the four facets and total scores were calculated [66].

### Neuropsychological measures (Detailed descriptions available in Text S1)

#### Digit Span – Backward [63]

The Digit Span – Backward is a verbal subtest of the WAIS-R used to measure of verbal working memory [67]. The raw score was used as the dependent variable.

#### Spatial Alternation Task [47]

This task assesses the alteration of motor responses to spatial locations on the basis of reinforcement information. Two red cars appeared on either side of the computer screen on each trial. The participant had to learn that the side on which the £20 note was located was being alternated after each correct response. The dependent variable was the number of errors committed before achieving 12 consecutive correct responses.

#### Probabilistic Response Reversal Task [54]

In the acquisition phase, the task assesses the ability to learn stimulus-response associations and, in the reversal phase, the task assesses the ability to alter stimulus-response associations as a function of contingency change. The reinforcement contingencies were probabilistic: the ‘correct’ pair was not always rewarded and the ‘incorrect’ pair was not always punished. There were two test pairs that changed contingency (reversing pairs) and four ‘dummy’ or non-reversing pairs. The two reversing pairs had the following probabilistic contingencies: 100–0; 80–20. The dependent variable was the number of errors committed before reaching the learning criterion of eight consecutive correct responses. If the participants did not meet the learning criterion, total errors made were analysed.

#### CGT [68]

On each trial, the participant was given 100 points and presented with a row of 10 boxes across the top of the screen, some of which were red and some of which were blue. The ratio of red:blue boxes varied from 1∶9 to 9∶1 in a pseudo-random order. The participant was instructed that the computer had hidden a token in one of the boxes, and that they must guess whether the token had been hidden in of the red or one of the blue boxes. On each trial, the participant first selected the colour (decision stage) and then betted a proportion of his total points on his colour decision (gambling stage). Each bet was presented for 2.5 seconds and offered in descending (95%, 75%, 50%, 25%, 5% of current points) or ascending (5%, 25%, 50%, 75%, 95% of the current points) sequences. After the bet was placed, the hidden token was revealed and the bet was added to or subtracted from the total score. The five principal dependent measures were: (1) Deliberation Time defined as the mean latency in milliseconds from presentation of the coloured boxes to the participant's response; (2) Quality of Decision-Making defined as the proportion of trials on which the participant chose to gamble on the more likely outcome, i.e. the colour of the greatest number of boxes; (3) Risk Taking defined as the percentage of the current points that the participant bet. To maintain the independence of betting behaviour and choice behaviour, analyses were limited to the trials where the participants selected the colour of the majority of boxes, i.e. trials on which they had more chance of winning than losing; (4) Risk Adjustment was defined as the degree to which a participant varies their risk-taking in response to the ratios of red to blue boxes within each trial; and (5) Delay Aversion was defined as the difference between risk-taking scores in the descending and the ascending conditions. High bets in both ascending and descending conditions reflect genuine risk-taking behaviour, whereas betting early in both the ascending and descending conditions reflects impulsivity (the participant does not wait for the bet to increase in the ascending condition or to decrease in the descending condition).

#### Passive Avoidance Learning Task [36]

The goal was to learn to respond to stimuli that lead to reward and to avoid responding to stimuli that lead to punishment. The participant was presented with 10 blocks of eight trials of distinct number identity. Each number was presented once during a block. Four numbers were associated with punishment (the S –) and four with reward (the S +). Participants were randomly assigned to one of two versions of the task: the numbers that were the S+ and the S – for one task were the S – and the S + in the other task, respectively. Reinforcement values were plus or minus 1, 700, 1400, and 2000 points for the four different S +/S –. The dependent variables were the number of passive avoidance (commission) errors (i.e., when participants approached a S−) and the number of omission errors (i.e., when the participants did not approach a S+).

#### Ethics statement

The study was approved by the Joint South London and Maudsley and the Institute of Psychiatry NHS Research and Ethics Committee (reference 06/Q0706/87).

### Procedure and apparatus

At the first interview, the study was fully explained both verbally and in writing to potential participants. After all of their questions were answered, participants signed consent forms. All potential participants who declined to participate or otherwise did not participate were not disadvantaged in any other way by not participating in the study. Participants included in the study were reimbursed at minimum hourly wage for each hour of testing completed. Participants were strongly encouraged to desist from using substances two weeks prior to participation and during the period of testing.

After all diagnostic interviews were completed, an appointment was scheduled for neuropsychological testing. Participants were reminded not to use drugs/alcohol prior to testing and that on arrival at the laboratory saliva and urine samples would be taken. Each participant was tested individually in a quiet interview room. The computer administered tasks were presented on a Dell Inspiron 510 m Laptop computer with a 15-in. (38.1 cm) colour monitor with participants seated about 0.5 m from the computer.

### Data analytic strategy

Data for quality of decision-making on the CGT were highly negatively skewed, with many participants selecting the likely outcome on the large majority of the trials. Normality could not be achieved using an arcsin transformation, thus data for quality of decision-making were analysed using non-parametric Kruskal –Wallis tests in each condition (ascending versus descending), collapsing across box ratio, and in each ratio (9∶1, 8∶2, 7∶3, 6∶4) collapsing across conditions. Significant between-group effects were followed-up using pair-wise post-hoc tests. Deliberation time data were positively skewed, with many participants responding quickly. The distribution of these values was successfully normalised using a logarithmic (log-10) transformation [69]. Data presented in the tables and figures are untransformed.

Continuous variables that conformed to parametric assumptions were analysed using Student's *t*-test, univariate analysis of variance (ANOVA) or repeated measures ANOVA. The Welch *t*' and *F*' tests and the Greenhouse-Geisser correction were applied where assumptions about homogeneity of variance and sphericity were violated, respectively [69]. Significant between-group effects were followed-up using pair-wise comparisons with Fisher's LSD procedure, which is the most powerful technique for post-hoc tests involving three groups [69], [70]. Effect sizes are reported as partial eta-squared (η_p_^2^; small ≥.01, medium ≥.06, large ≥.14) [69]. Categorical variables were analysed using Chi square tests. Results were considered statistically significant at *p*<.05, two-tailed. Not all the participants completed all the tasks, so degrees of freedom vary slightly across analyses.

## Results

### Final sample

The characteristics of the three groups are reported in Table 1. The three groups were similar with respect to age, IQ, and ethnicity. As intended, there were significant differences between all three groups on total and 4-facet PCL-R scores. The non-offenders additionally differed from the offenders by presenting significantly fewer conduct disorder symptoms prior to age 15, lower scores for proactive and reactive aggression, and less substance misuse. Consistent with previous studies, the ASPD+P offenders, as compared to the ASPD−P offenders, presented significantly more symptoms of conduct disorder prior to age 15, were significantly younger at first conviction for a violent offence, obtained higher scores for proactive aggression and similar scores for reactive aggression, and there was a trend suggesting that they had accumulated more convictions for violent crimes and they had higher scores for proactive aggression. The proportions of ASPD+P and ASPD−P offenders with substance use disorders were similar.

**Table 1:** Comparisons of Sociodemographic, Clinical, and Behavioural Characteristics of Non-offenders, Violent Offenders with ASPD−P, and Violent Offenders with ASPD+P.

Measure	Non-offenders (n = 21)	ASPD–P (n = 28)	ASPD+P (n = 17)	Group differences
Age in years	35.0 (8.2)	35.8 (8.4)	40.0 (9.0)	*F*(63) = 1.87
Full Scale IQ	95.1 (11.0)	91.9 (10.2)	88.9 (9.9)	*F*(63) = 1.72
% Caucasian	61.9	67.9	41.2	*χ^2^*(2) = 3.22
% with PD other than ASPD				
Cluster A	0	10.7	17.6	*χ^2^*(2) = 3.69
Cluster B	0	14.3	23.5	*χ^2^*(2) = 5.1#
Cluster C	0	7.1	11.8	*χ^2^*(2) = 2.38
PCL–R total	3.8 (2.8)^a^	16.7 (4.1)^b^	28.3 (2.1)^c^	*F*(63) = 260.73***
PCL–R Interpersonal facet	0.4 (1.0)^a^	1.7 (1.4)^b^	3.9 (1.3)^c^	*F*(63) = 36.14***
PCL–R Affective facet	0.6 (0.9)^a^	2.9 (1.8)^b^	6.2 (2.9)^c^	*F*(63) = 63.37***
PCL–R Lifestyle	1.9 (1.5)^a^	5.1 (2.1)^b^	6.9 (1.8)^c^	*F*(63) = 34.91***
PCL–R Antisocial	0.3 (0.6)^a^	5.8 (2.1)^b^	8.6 (1.4)^c^	*F*(63) = 134.60***
CD symptoms Counts	0.7(1.2)^a^	4.4 (2.8)^b^	7.6 (3.4)^a^	*F*(63) = 32.60***
Age at first violent convictions	n/a	23.4 (8.1)^a^	16.8 (3.3)^b^	*t*' (43) = −3.75**
Number of violent convictions	n/a	4.7 (3.4)	6.9 (5.2)	*t*(43) = 1.70#
RPAQ Aggression total	7.3 (3.4)^a^	17.4 (9.1)^b^	22.3 (11.3)^b^	*F*(62) = 15.90***
Proactive aggression	2.3 (3.3)^a^	8.4 (5.4)^b^	12.5 (7.1)^c^	*F*(62) = 17.66***
Reactive aggression	4.9 (3.1)^a^	9.0 (5.8)^b^	11.5 (6.5)^b^	*F*(62) = 7.49**
% Alcohol				
Abuse	11.8	25.0	26.7	*χ^2^*(2) = 1.39
Dependence	5.9^a^	39.3^b^	26.7^b^	*χ^2^*(2) = 6.04*
% Cannabis				
Abuse	5.9	14.3	13.3	*χ^2^*(2) = .78
Dependence	11.8	32.0	25.0	*χ^2^*(2) = 2.27
% Cocaine				
Abuse	0	0	6.7	*χ^2^*(2) = 3.05
Dependence	0	20.0	25.0	*χ^2^*(2) = 4.48
% Stimulants				
Abuse	0	3.6	0	*χ^2^*(2) = 1.16
Dependence	0	4.2	0	*χ^2^*(2) = 1.23
% Sedatives				
Abuse	0	3.6	0	*χ^2^*(2) = 1.16
Dependence	0	4.0	0	*χ^2^*(2) = 1.18
% Opioid				
Abuse	0	3.6	13.3	*χ^2^*(2) = 3.21
Dependence	0	12.0	8.3	*χ^2^*(2) = 2.14
% Hallucinogenics				
Abuse	0	7.1	0	*χ^2^*(2) = 2.37
Dependence	0	0	0	n/a

*Note*. Unless otherwise stated, means are presented with standard deviations in parentheses for each group. Means with different superscripts within each row indicate a significant difference. PD  =  Personality Disorder; ASPD–P  =  Antisocial Personality Disorder without Psychopathy; ASPD+P  =  Antisocial Personality Disorder with Psychopathy; n/a  =  Not Applicable; PCL–R  =  Psychopathy Checklist – Revised (Hare, 2003); RPAQ  =  Reactive Proactive Aggression Questionnaire (Raine et al., 2006). One offender with ASPD–P did not complete the RPAQ Aggression Questionnaire.

# *p*<.10. ** *p*<.01. *** *p*<.001.

### Digit Span – Backward

In line with the a priori hypothesis, there was a statistically significant group difference in scores on the Digit Span-Backward task, *F*' (2, 37.15)  = 3.57, *p* = .038, η_p_^2^  = .11. Post-hoc tests indicated that the non-offenders (*M* = 7.33, *SD*  = 3.76) repeated more digits than the ASPD+P (*M* = 4.69, *SD*  = 2.21; *p* = .009) and the ASPD−P group, but only at a trend level (*M* = 5.75, *SD*  = 2.62; *p* = .07). Scores for the two ASPD groups did not differ (*p* = .26).

### Spatial Alternation Task

No participant failed the task. Contrary to the a priori hypothesis, scores for the three groups on the Spatial Alteration Task did not differ, *F*(2, 62)  = .36, *p* = .70, η_p_^2^  = .01 (ASPD+P: *M* = 3.12, *SD*  = 2.52; ASPD−P: *M* = 3.96, *SD*  = 7.13; non-offenders: *M* = 4.52, *SD*  = 3.16). Five participants (one ASPD+P, one ASPD−P and two non-offenders) were identified as outliers with respect to their groups, but removing them from the analyses did not alter the pattern of results, *F*(2, 57)  = 1.38, *p* = .26, η_p_^2^  = .05.

### Probabilistic Response Reversal Task

All participants reached the learning criterion for the acquisition and reversal phases of the pair with the 100–0 contingency. However, three non-offenders failed to reach the criterion for the acquisition of the pair with the 80–20 contingency. In line with Budhani et al. [54], their data were excluded from the analyses since it was unclear if these participants had learned the stimulus-response association so that response reversal could be examined. In addition, data from one ASPD+P and three ASPD−P offenders and three non-offenders were excluded from the analyses as their scores were more than 2.5 standard deviations above their respective group means.

**Figure 1:**
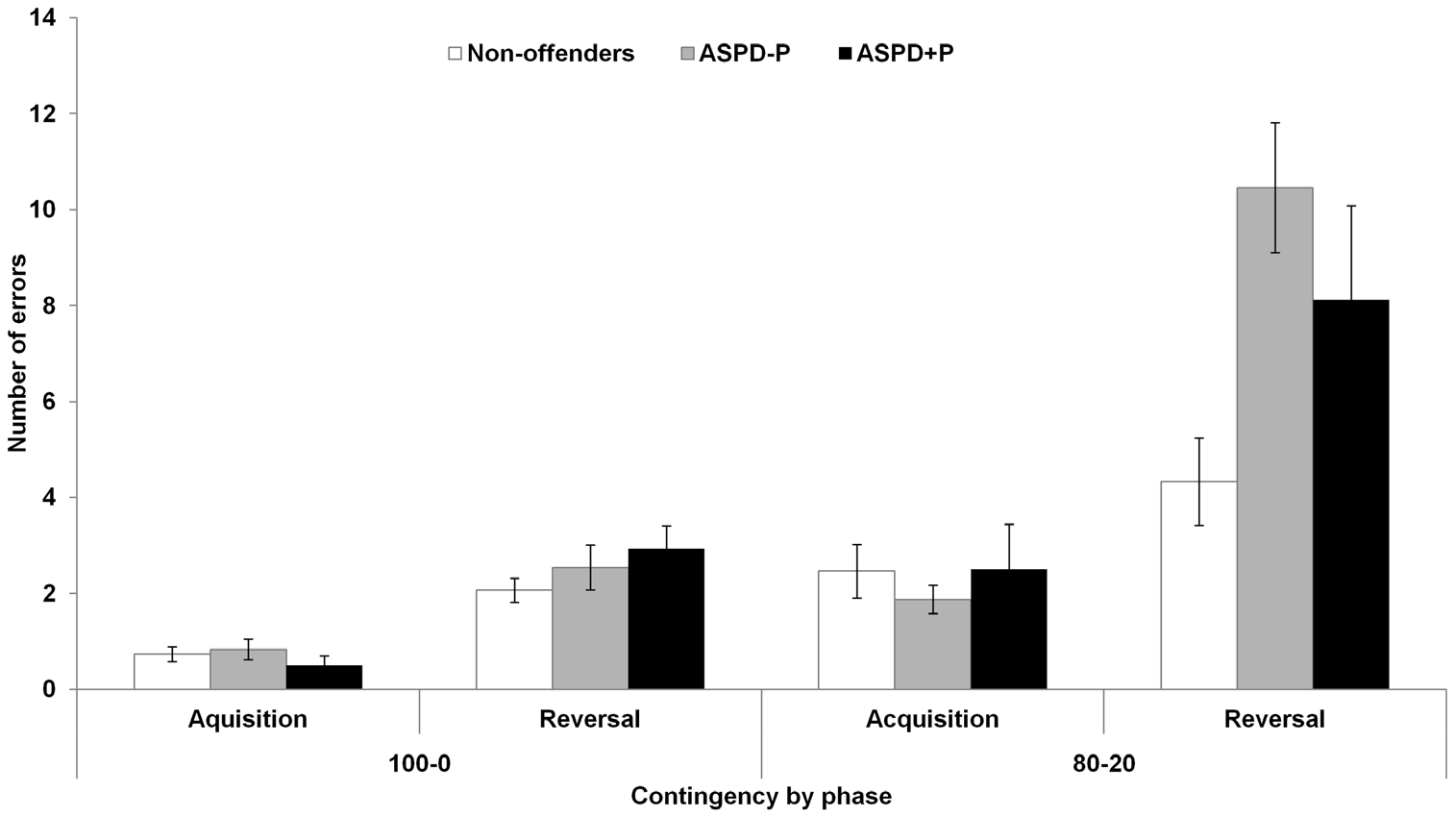
Performance of the three groups on the Probabilistic Response Reversal Task as indicated by the number of errors to criterion made in the acquisition and reversal phases of the pair 100–0 (left) and of the pair 80–20 (right). Maximum errors  = 40. Error bars indicate standard error of the mean. ASPD–P  =  Antisocial Personality Disorder without Psychopathy; ASPD+P  =  Antisocial Personality Disorder with Psychopathy.

A 3 (group: ASPD+P, ASPD−P, non-offenders) ×2 (contingency: 100–0 versus 80–20) ×2 (phase: acquisition versus reversal) mixed model ANOVA revealed that there was a statistically significant main effect of group, *F*(2, 52)  = 3.94, *p* = .03, η_p_^2^  = .13. Post-hoc tests indicated that men with ASPD−P (*M* = 15.7, *SD*  = 6.7; *p* = .01) and those with ASPD+P, albeit at a trend level (*M* = 14.1, *SD*  = 8.6; *p* = .07), committed more errors than the non-offenders (*M* = 9.6, *SD*  = 3.5). Scores of ASPD+P and ASPD−P offenders did not differ (*p* = .45). There was a highly significant main effect of phase, *F*(1, 52)  = 51.64, *p*<.001, η_p_^2^  = .50, indicating that participants committed more errors during the reversal phase (*M* = 10.6, *SD*  = 6.9) than the acquisition phase (*M* = 2.9, *SD*  = 2.7) (Figure 1). In addition, there was a highly significant main effect of contingency, *F*(1, 52)  = 49.23, *p*<.001, η_p_^2^  = .49. As can been seen from Figure 1, participants committed more errors on the stimulus pair with a 80–20 contingency (*M* = 10.3, *SD*  = 6.8) than on the stimulus pair with a 100–0 contingency (*M* = 1.8, *SD*  = 0.2). There was also a significant phase by contingency interaction, *F*(1, 52)  = 10.84, *p* = .002, η_p_^2^  = .17. Importantly, there were also a significant group by phase interaction, *F*(2, 52)  = 58.38, *p* = .02, η_p_^2^  = .15, and a significant group by phase by contingency interaction, *F*(1, 52)  = 49.23, *p* = .049, η_p_^2^  = .11. While the three groups committed a similar number of errors for the two contingency pairs in the acquisition phase and the reversal phase of the 100–0 contingency pair, in comparison to the non-offenders (*M* = 4.3, *SD*  = 3.5), offenders with ASPD−P (*M* = 10.5, *SD*  = 6.7; *p* = .005) and those with ASPD+P, albeit at a trend level (*M* = 8.1, *SD*  = 7.8; *p* = .10), committed more errors on the reversal phase of the of the 80–20 contingency pair (Figure 1). The comparison between ASPD+P and ASPD−P was not significant (*p* = .26).

### CGT

#### Deliberation time

**Figure 2:**
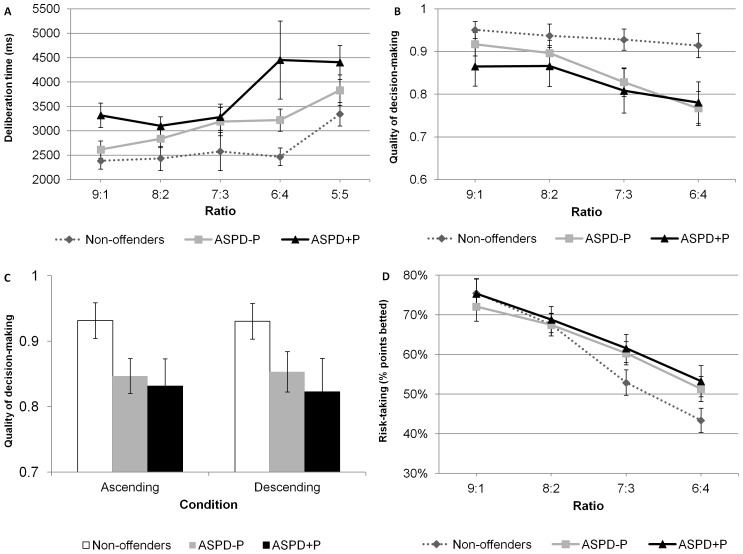
Performance of the three groups on the CGT as indicated by the deliberation time by ratio (top left), quality of decision-making by ratio (top right), quality of decision-making by condition (bottom left), risk-taking by ratio (bottom right). Error bars indicate standard error of the mean. ASPD–P  =  Antisocial Personality Disorder without Psychopathy; ASPD+P  =  Antisocial Personality Disorder with Psychopathy.

A 3 (group: ASPD+P, ASPD−P, non-offenders) ×4 (ratio: 9∶1, 8∶2, 7∶3, 6∶4) mixed model ANOVA on deliberation time indicated that participants took more time to make decisions on trials with less favourable ratios as indicated by a significant main effect of ratio, *F*(3, 189)  = 6.38, *p*<.001, η_p_^2^  = .09 (Figure 2). There was a statistically significant main effect of group, *F*(2, 63)  = 5.69, *p* = .005, η_p_^2^  = .15. Both offenders with ASPD+P (*M* = 3629.6, *SD*  = 1285.5; *p* = .001) and those with ASPD−P (*M* = 3060.5, *SD*  = 1103.2; *p* = .04) took more time to make decisions than the non-offenders (*M* = 2571.7, *SD*  = 991.0). In addition, there was a significant group by ratio interaction, *F*(6, 189)  = 3.53, *p* = .002, η_p_^2^  = .10. There were differences in deliberation time between some of the ratios among men with ASPD+P (9∶1 vs. 7∶3, *p* = .009; 9.1 vs. 6∶4, *p* = 004) and among those with ASPD−P (6∶4 vs.7∶3, *p* = .008; 6∶4 vs. 8∶2, *p* = .001; 6∶4 vs. 9∶1, *p* = .048), but this was not observed for the non- offender group (all *p*s >.69).

#### Quality of decision-making

There were statistically significant group differences in the quality of decision-making in both the ascending, *χ^2^* (2)  = 6.74, *p* = .034, η_p_^2^  = .10, and the descending, conditions, *χ^2^* (2)  = 9.42, *p* = .009, η_p_^2^  = .14. Follow-up post-hoc tests for the ascending condition indicated significant differences between the non-offenders and both the ASPD−P (*p* = .02) and ASPD+P offenders (*p* = .04). No differences were detected in scores of the two ASPD groups (*p* = 1) (Figure 2). Similarly, follow-up post-hoc-tests for the descending condition revealed significant differences between the non-offenders and both the ASPD+P (*p* = .01) and ASPD−P offenders (*p* = .01), while the two ASPD groups did not differ from each other (*p* = .72). The quality of decision-making did not differ across groups for the ratios 9∶1, *χ^2^* (2)  = 3.61, *p* = .17, η_p_^2^  = .05, and 8∶2, *χ^2^* (2)  = 1.14, *p* = .57, η_p_^2^  = .02, but there were statistically significant group differences in the quality of decision-making for the less favourable ratios 7∶3, *χ^2^* (2)  = 7.33, *p* = .03. η_p_^2^  = .11, and 6∶4, *χ^2^* (2)  = 8.98, *p* = .01, η_p_^2^  = .14. Post-hoc tests for the ratio 7∶3 revealed that, while the there was no difference between the two ASPD groups (*p* = .97), there was a significant difference between the non-offenders and both the ASPD−P offenders (*p* = .01) and ASPD+P offenders (*p* = .03). A similar pattern was observed for the ratio 6∶4: while the there was no difference between the two ASPD groups (*p* = .87), there was a significant difference between the non-offenders and both the ASPD−P offenders (*p* = .01) and ASPD+P offenders (*p* = .02).

#### Risk-taking, risk adjustment, and delay aversion

A 3 (group: ASPD+P, ASPD−P, non-offenders) ×4 (ratio: 9∶1, 8∶2, 7∶3, 6∶4) ×2 (condition: ascending versus descending) ANOVA on risk-taking identified a significant main effect of condition, *F*(1, 61)  = 90.48, *p*<.001, η_p_^2^  = .60, and of ratio, *F*(1.52, 92.41)  = 83.13, *p*<.001, η_p_^2^  = .58. (The degree of freedom for the repeated ANOVA is 61 instead of 63 because the risk-taking measure could not be calculated for one ASPD+P and one ASPD−P, as they bet on the colour in the minority (i.e. the less likely outcome) – see description of how Risk-taking is calculated in Text S1.) Participants bet more on the descending condition and less as the ratio of boxes became less favourable (Figure 2). The main effect of group and the interactions terms were not statistically significant (all *F*s <2.23). A one-way ANOVA on the risk adjustment measure indicated that there was a trend for a group difference, *F*(2, 63)  = 2.97, *p* = .058, η_p_^2^  = .60, suggesting that the two ASPD groups adjusted their betting less than the non-offenders (Figure 2). Finally, there was no main effect of group on the delay aversion measure, *F*(2, 61)  = .12, *p* = .99, η_p_^2^  = .00, indicating no group difference in impulsivity.

### Passive Avoidance Learning Task

**Figure 3:**
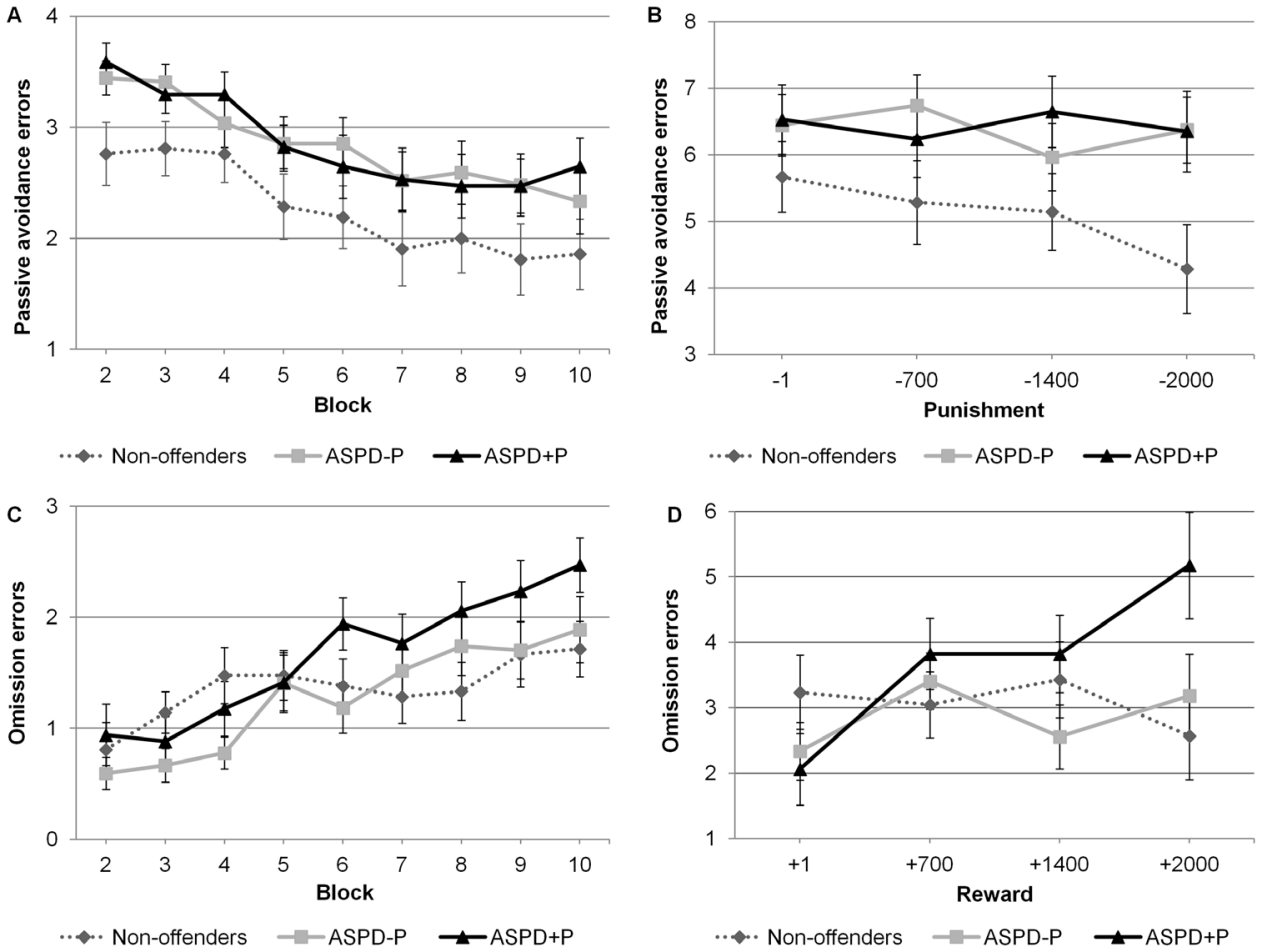
Performance of the three groups on the Passive Avoidance Learning Task as indicated by the number of passive avoidance errors by block (top left), number of passive avoidance errors by punishment levels (top right), number of omission errors by block (bottom left), and number of omission errors by reward levels (bottom right). Error bars indicate standard error of the mean. ASPD–P  =  Antisocial Personality Disorder without Psychopathy; ASPD+P  =  Antisocial Personality Disorder with Psychopathy.

Following R.J.R. Blair et al [36], each initial presentation of a stimulus was treated as a learning trial, so results from the first block were omitted from the analysis. A 3 (group: ASPD+P, ASPD−P, non-offenders) ×4 (punishment values: 1, 700, 1400, 2000) ×9 (blocks) model ANOVA was performed on the number of commission errors. There was a statistically significant main effect of block, *F*(5.91, 366.63)  = 13.67, *p*<.001, η_p_^2^  = .18, indicating a decrease in the number of commission errors as the task progressed (Figure 3). The main effect of group fell short of statistical significance, *F*(2, 62)  = 2.92, *p* = .06, η_p_^2^  = .09, suggesting that the two ASPD groups tended to make more commission errors than the non-offenders. There was no statistically significant main effect of punishment or interaction effects (all *Fs* <1.1).

A 3 (group: ASPD+P, ASPD−P, non-offenders) ×4 (reward values: 1, 700, 1400, 2000) mixed model ANOVA conducted on the omission errors revealed a main effect of reward, *F*(2.47, 153.26)  = 2.94, *p* = .045, η_p_^2^  = .05. As illustrated in Figure 3, participants made fewer omission errors for smaller reward values. There was also a main effect of block, *F*(5.25, 325.23)  = 15.83, *p*<.001, η_p_^2^  = .20. As can be seen from Figure 3, participants made more errors as the task progressed. While the main effect of group was not significant, *F*(2, 62)  = 1.17, *p* = .32, η_p_^2^  = .04, there was a significant group by reward interaction, *F*(4.94, 153.26)  = 2.65, *p* = .03, η_p_^2^  = .08. While the performance of the ASPD−P and the non-offender groups was not influenced by the level of reward, the ASPD+P offenders committed fewer errors at the lowest level of reward (+1) as compared to levels +700 and +2000 (*ps*  = .018 and .008, respectively). The group by block interaction was not statistically significant, *F*(10.49, 325.25)  = 1.69, *p* = .08, η_p_^2^  = .05.

## Discussion

The present study is the first to use a comprehensive battery of neuropsychological tests to assess cool and hot EF, comparing two groups of violent offenders and one group of healthy non-offenders. A summary of the results is presented in Table 2. As hypothesized, both the violent offenders with ASPD+P and those with ASPD−P showed similarly poor performance on the Digit Span – Backward test assessing cool EF, and on several tests of hot EF as compared to healthy non-offenders. However, infirming our second hypothesis, the ASPD+P offenders did not make more commission errors than the ASPD−P offenders on the Passive Avoidance Learning Task. In fact, the performance of the two groups of violent offenders did not differ on any of the tasks.

**Table 2:** Summary of Task Performance of Non-offenders, Violent Offenders with ASPD−P, and Violent Offenders with ASPD+P.

Neuropsychological measure	Group and Interaction Effects	Post-hoc†
Digit Span – Backward	Group	Non-offenders > ASPD+P, ASPD−P#
Spatial Alternation Task	–	–
Passive Avoidance Learning Task		
Commission errors	Group#	Non-offenders > ASPD+P#, ASPD−P#
Omission errors	–	–
Probabilistic Response Reversal	Group	Non-offenders > ASPD+P#, ASPD−P
Acquisition errors	–	–
Reversal errors 80–20 pair	Group x Phase	Non-offenders > ASPD+P#, ASPD−P
Cambridge Gamble Task		
Deliberation time	Group	Non-offenders > ASPD+P, ASPD−P
Quality of decision-making	Group ↑↓	Non-offenders > ASPD+P, ASPD−P
	Group ratio 7∶3	Non-offenders > ASPD+P, ASPD−P
	Group ratio 6∶4	Non-offenders > ASPD+P, ASPD−P
Risk-taking	–	–
Risk adjustment	Group#	Non-offenders > ASPD+P#, ASPD−P#
Delay aversion	–	–

*Note*. Better performance > worse performance.

† The performance of the ASPD+P and ASPD−P did not differ on any of the tasks.

# Trend for group difference.

– No statistically significant group difference.

↑ Ascending condition.

↓ Descending condition.

Importantly, violent offenders with a life-long history of antisocial and aggressive behaviour as compared to non-offenders matched for age, IQ and ethnicity, showed deficits in an array of both cool and hot EF. Both offenders with ASPD+P and those with ASPD−P showed impaired performance on the Digit Span – Backward, a measure of working verbal memory indexing cool EF. Impaired verbal working memory limits reflection during problem solving, particularly in situations requiring adaptive social responses [7], [71], [72]. While a previous study reported that offenders with ASPD+P performed similarly to offenders without psychopathy on this task [53], the results of the present study show that, when compared to healthy non-offenders, both offenders with ASPD+P and those with ASPD−P display deficits in verbal working memory as do persistently aggressive children and adults [11], [72]. Interestingly, two previous studies did not find a verbal working memory deficit among men with ASPD−P with no history of criminality or substance misuse [43], [73]. Taken together, the results of the present study and the extant literature may be interpreted to suggest that impaired verbal working memory is associated with a life-long pattern of aggressive behaviour.

While both groups of violent offenders showed poorer verbal working memory than the non-offenders, on another test of cool EF, the Spatial Alternation Task, they showed no impairment. This finding is consistent with results of the only previous study to assess offenders with this task in which offenders with and without psychopathy performed similarly [47]. Our results extend the previous findings by showing that, while the role of the DLPFC in the alteration of motor responses to spatial locations on the basis of reinforcement information is not impaired in offenders with ASPD+P and those with ASPD−P, they do exhibit impairments in verbal working memory, another cool EF subsumed by the DLPFC.

The two groups of violent offenders also showed impairments in hot EF as compared to the non-offenders. In the reversal phase of the Probabilistic Response Reversal Task, offenders with ASPD−P committed significantly more errors than the non-offenders in the condition where the stimulus-response association was less clear (i.e., 80–20 contingency pair). The offenders with ASPD+P showed a trend in the same direction. A previous study of offenders using the same paradigm reported that those with psychopathy, as compared to those without psychopathy, committed more errors on the reversal phase of the 100–0 and 80–20 pairs [54]. However, the finding that the ASPD−P offenders showed impairments on the response reversal task is consistent with a previous study [74] showing that offenders with moderate PCL-R scores (between 21–29; insufficient to warrant a diagnosis of psychopathy in the U.S.) were impaired in response reversal in comparison to offenders without psychopathy (PCL-R scores range: 0–20).

On the CGT, the two groups of offenders, as compared to the non-offenders, also displayed poorer quality of decision-making despite increased deliberation times and a strong trend for less modulation of their betting as the probability of loss increased, but similar levels of impulsivity and risk-taking. The two groups of violent offenders, like the non-offenders, deliberated longer before making a decision as the box ratio became less favourable, thereby showing an understanding of the trial-by-trial probabilities and of the increased risk of losing points. This pattern of results – long delay and poor decision making - resembles that observed among patients with lesions in the VMPFC (e.g., [68], [75]; but see [76]). Thus, although they were aware of the increased risk of loss, the offenders failed to adjust their behaviour to the increasing risk of losing points, just as they persist in engaging in antisocial behaviour despite knowing that it will likely lead to negative consequences.

Perhaps one of the most novel aspects of this study are the results of the delay aversion and risk taking measures, which indicate that the two groups of ASPD offenders were no more impulsive or risk-taking (at least not at the two most favourable ratios) than the non-offenders. These results are likely due to the fact that the CGT is a decision-making task in which outcome probabilities and the associated risks are explicit. By contrast, the few studies that have examined affective decision-making of men with ASPD+P or ASPD−P and shown increased risk-taking behaviour used the Iowa Gambling Task in which outcome probabilities are unknown. This latter task relies on the integrity and coordination of several processes, including stimulus-reinforcement learning, reversal learning, set-shifting, and working memory [77]. Since men with ASPD, regardless of psychopathy scores, are known to be impaired on some of these processes, this might explain results of previous studies of risk-taking on the Iowa Gambling Task. Much evidence indicates that men with ASPD, regardless of psychopathy, show impulsive behaviour in the form of impaired response inhibition (e.g., [50], [57]). Results of the present study suggest that they may not display impulsivity defined as delay aversion. A previous study examined delay aversion among offenders with and without psychopathy [78]. Low anxious psychopaths, in comparison to low anxious non-psychopaths, delayed gratification less often in the condition that involved rewards and punishments, but not in the condition that involved rewards only. Clearly, additional research examining different forms of impulsivity in relation to ASPD and psychopathy is warranted.

On another test of hot EF, the Passive Avoidance Learning Task, the violent offenders were impaired as compared to the non-offenders. There was a trend (*p* = .06) indicating that the two offender groups made more commission errors than the non-offenders, but, contrary to our second hypothesis, no evidence of an increased number of commission errors in the ASPD+P group compared to the ASPD−P group. As hypothesized, there was no evidence of a group difference in omission errors. These results show that this failure to learn from punishment cues characterizes not only violent offenders with ASPD+P, but also those with ASPD−P. The results of the present study suggest that both violent offenders with ASPD+P and those with ASPD−P have difficulty in stimulus-punishment associations.

Violent offenders with ASPD, both those with and without additional diagnoses of psychopathy, showed impairments in verbal working memory, and in adaptive affective decision-making. They failed to learn from punishment cues, to change their behaviour in the face of changing contingencies, and made poorer quality decisions despite longer periods of deliberation before such decisions. The combination of these impairments may go some way towards explaining why violent offenders with ASPD with and without psychopathy are characterized by irresponsibility, recklessness, persistent aggressive behaviour, and engagement in multiple other types of antisocial behaviours despite knowing that such behaviour will likely lead to negative consequences for themselves and/or others [14].

These findings need to be replicated. The absence of statistically significant differences in performance on any of the neuropsychological tasks between the violent offenders with ASPD+P and those with ASPD−P suggests shared deficits in cool and hot EF, at least based on the tasks used and the processes they index. These results are consistent, however, with our structural brain imaging findings on an overlapping sample showing differences between violent offenders with ASPD+P and those with ASPD−P in gray matter volume of the superior/medial prefrontal cortex and temporal poles, but no differences in gray matter volume the amygdala, VMPFC or DLPFC [23]. Additionally, reduced fractional anisotropy in the right uncinate fasciculus (the primary white matter tract connecting the VMPFC and the anterior temporal lobes) has been demonstrated in both violent men with ASPD+P [79], [80] and those with ASPD−P [81]. Thus, the results from the present study are consistent with this emerging evidence from brain imaging studies and might reflect that fact that, while the offenders with ASPD+P scored twice as high as the offenders with ASPD−P on the PCL-R facet 1 and facet 2 indexing the core interpersonal and affective features of the syndrome of psychopathy, these two groups of violent offenders share many characteristics, most importantly a childhood onset of conduct problems that persist into adulthood and violent behaviour. The present results suggest that both offenders with ASPD+P and those with ASPD−P present similar EF impairments despite differences in the types of aggressive behaviour in which they engage, personality traits, emotion processing, and structural and functional brain anomalies.

Several methodological limitations should be considered in interpreting the results of the present study. One, there may have been a lack of statistical power to detect group differences resulting from the relatively small number of violent offenders with ASPD+P. The number of participants, however, was similar to many of the previous neuropsychological studies in the field (e.g., [51], [54], [82]). Two, the use of the validated PCL-R cut-off score for European offenders to identify the syndrome of psychopathy may have lessened the likelihood of observing cognitive impairments. However this is unlikely as the pattern of results generally showed that impairments previously reported as characterizing offenders with psychopathy also characterized those with ASPD−P. Three, as is evident from the review of the literature and the present results, findings about psychopathy depend to a large extent on the comparison group used in each study. Therefore, all analyses were re-run excluding six offenders with ASPD−P whose total PCL-R scores were between 24 and 20. Again, no significant group differences between offenders with ASPD+P and ASPD−P were found. Four, the violent offenders with ASPD, like almost all people with ASPD [83], had a history of substance misuse. While objective tests assured that the participants were not tested when intoxicated, it is possible that past substance misuse led to some of the deficits in performance that were observed. Finally, the use of digits as central stimuli in two of the tasks (i.e., the Digit Span – Backward and the Passive Avoidance Learning Task) may not have been ideal for testing individuals with low levels of education.

This study also has several strengths. One, it is the first study to directly contrast various aspects of hot EF in violent offenders with ASPD+P and violent offenders with ASPD−P and to compare test performance to that of healthy non-offenders. Two, this is the first study to include offenders who were convicted of several violent crimes, diagnosed by forensic psychiatrists using standardized, validated interview protocols, and examined using a comprehensive battery of neuropsychological tests that assessed both cool and hot EF. Three, this study was the first to examine affective decision-making under risk among men with ASPD+P and men with ASPD−P. Finally, the three groups did not differ in terms of age, IQ, and ethnicity.

The findings from this study provide novel evidence that, in comparison to healthy non-offenders, violent offenders with ASPD+P and violent offenders with ASPD−P present impairments in both cool and hot EF such as verbal working memory, response reversal, affective decision-making under risk, and stimulus-reinforcement-based decision-making. The performance of the two groups of offenders on these tasks did not differ suggesting shared deficits in EF, at least based on the tasks used and the processes they index. The combination of these impairments may help to explain why violent offenders with ASPD, both those with and without psychopathy, persist in engaging in antisocial behaviours despite knowing the risks of negative consequences to themselves and/or others [14]. Crucially, given the differences in their responses to cognitive-behavioural rehabilitation programs aimed at reducing violence and recidivism [12], [13], additional research is needed to further understanding of the neurobiological and psychological similarities and differences in these two types of offenders. Functional magnetic resonance imaging, which has never been used to directly compare these two groups of violent offenders, would be highly informative in this regard since it can detect subtle alterations in neural processing that may not be observable with behavioural indices.

## Supporting Information

Text S1 **Supporting text.**
(DOCX)
