# Supplementary material for: Cool and Hot Executive Function Impairments in Violent Offenders with Antisocial Personality Disorder with and without Psychopathy
Source: PLoS One. 2013 Jun 20;8(6):e65566. doi: 10.1371/journal.pone.0065566 (PMC3688734; doi:10.1371/journal.pone.0065566)
Supplement: Text S1 — Supporting text. (DOCX) [file pone.0065566.s001.docx]

**Supplementary Material for:**

Cool and Hot Executive Function Impairments in Violent Offenders with Antisocial Personality Disorder With and Without Psychopathy

Stéphane A De Brito ^1^, Essi Viding ^2^, Veena Kumari ^3^, Nigel Blackwood ^4^, Sheilagh Hodgins ^4, 5^

^1^ School of Psychology, University of Birmingham, Birmingham, United Kingdom.

^2^ Division of Psychology and Language Sciences, University College London, United Kingdom.

^3^ Department of Psychology, Institute of Psychiatry, King’s College London, United Kingdom.

^4^ Department of Forensic and Neurodevelopmental Science, Institute of Psychiatry, King’s College London, United Kingdom.

^5^ Département de Psychiatrie Université de Montréal, Québec, Canada.

Correspondence:

Stéphane A. De Brito, School of Psychology, University of Birmingham, B15 2TT, Birmingham, United Kingdom.Tel: +44(0)121 414 2641. E-mail: [s.a.debrito@bham.ac.uk](mailto:s.a.debrito@bham.ac.uk)

**Introduction**

To examine cool executive function (EF), verbal working memory and the alteration of motor responses to spatial locations were assessed using the Digit Span Backward subtest of the Wechsler Adult Intelligence Scale [1] and the Spatial Alternation Task [2], respectively. These two abilities have been shown to be disrupted following lesions to the dorsolateral prefrontal cortex (DLPFC) [3]. Impairments in verbal working memory have been found in physically aggressive children and adults [4]. It has been proposed that impaired verbal working memory may limit reflection during problem solving, particularly in situations requiring adaptive social responses [5-7]. It has also been suggested that spatial impairments, possibly indexing anomalies in right hemisphere affect regulation, might contribute to the development of persistent antisocial behaviour [8].

To assess hot EF, response reversal learning, decision-making under risk, and stimulus-reinforcement-based decision-making were examined. Reversal learning (i.e., the ability to alter behaviour as a function of change in reinforcement contingencies) was examined using the Probabilistic Response Reversal Task [9]. Impairment in reversal learning is a characteristic of patients with ventromedial prefrontal cortex (VMPFC) lesions [10]and it has been proposed to increase the risk for frustration-based reactive aggression [11]. Decision-making under risk (i.e., when outcome probabilities are known) was assessed using the Cambridge Gamble Task (CGT) [12]. Performance on this task is also impaired in patients with VMPFC lesions [12,13]. Importantly, the CGT presents several advantages over the Iowa Gambling Task (IGT) used in most previous studies, which have examined risk-taking in ASPD+P or ASPD-P [14,15]. First, in contrast to the IGT where outcome probabilities are unknown, the outcome probabilities in the CGT are known meaning that it does not simultaneously involve other processes such as stimulus-reinforcement learning, reversal learning, set-shifting and working memory [16]. Further, the CGT teases apart risk-taking and impulsive behaviours, two central features of both ASPD+P and ASPD-P. Finally, the CGT measures delay aversion (i.e., tendency to choose a small immediate reward over a delayed larger reward), one form of impulsivity that has not been studied in offenders with ASPD+P and those with ASPD-P. High delay aversion has been linked to poor self-regulation and to physical aggression [17-19]. The last hot EF task, the Passive Avoidance Learning Task [20], indexes stimulus-reinforcement-based decision-making, a form of decision-making disrupted by lesions of the ventromedial pathway connecting mesolimbic reward circuitry, including the amygdala, hippocampus, and striatum, to the VMPFC [21,22].

**Materials and Methods**

**Neuropsychological Measures**

**Digit Span – backward [1].** The examiner verbally presented digits at a rate of one per second and the participant was asked to repeat the digits in reverse order. The number of digits increased by one until the participant consecutively failed two trials each with the same number of digits.

**Spatial Alternation Task [2].** On the first trial of the task, both sides were baited with a £20 note. For the second trial, the £20 note was located on the side not chosen on the first trial. Following a correct response, the other side was baited on each subsequent trial. Following an incorrect response, the £20 note remained behind one side until the participant made a correct response. The time between the response and the next stimulus presentation was 1 second. The task ended when the participant made 12 consecutive correct responses (learning criterion) or 80 trials.

**Probabilistic Response Reversal [9].** Stimuli comprised 12 line drawings of animals, which were shaded in different colours. Stimuli measured 4 cm x 4 cm and were presented on a gray background. Stimuli were randomly paired at the beginning of the task. On each trial, a pair of stimuli was presented. Sixteen possible locations were randomly selected for each stimulus presentation. The participant was required to choose one of the stimuli by clicking on it with the mouse. Upon choosing they received either positive (‘you win 100 points’) or negative (‘you lose 100 points’) feedback depending on the reinforcement contingency of that pair. One of the animals in each pair was always more likely than the other to be rewarded rather than punished. The reinforcement contingencies of the reversing pairs remained constant for 40 trials (phase 1; acquisition of the discrimination). Upon completion of 40 trials the reinforcement contingency, the reversing pairs were reversed (phase 2; reversal of the discrimination). Thus, the previously correct stimulus became the incorrect stimulus and the previously incorrect stimulus became the correct stimulus. Three of the four non-reversing dummy pairs had the contingency 100–0, the fourth 80–20. The contingency of the non-reversing pairs remained the same for all 40 trials. Participants began the task with 0 points. A running total of points was visible at the bottom of the screen during feedback only. Trials were self-paced. All participants completed 270 trials regardless of performance.

**Cambridge Gamble Task [12].** The task was administered on a laptop with responses recorded via a touch-sensitive screen and was completed within 25 minutes. Relevant information was presented to the participants ‘up-front’ and there was no need to learn or retrieve information over consecutive trials. Participants completed four practice trials of the task, followed by four blocks of nine trials in the descending and the ascending conditions. On each trial, the participant was presented with a row of 10 boxes across the top of the screen, some of which were red and some of which were blue. The ratio of red to blue boxes varied from 1:9 to 9:1 in a pseudo-random order^1^. The participant was told that the computer had hidden a token in one of the boxes, and that they must guess where the token had been hidden. In the decision-making stage, the participant was asked to guess whether the yellow token was hidden in a red box or a blue box. This is a relatively simple probabilistic judgment in which the participant should choose the colour of the majority of the boxes. The participant responded by touching one of two coloured panels marked ‘Red’ or ‘Blue. In the gambling stage, participants began with 100 points and, on each trial, selected the proportion of these points that they wished to gamble. Each potential bet was displayed for 5 seconds before the next potential bet in the sequence appeared. The participant was asked to select any bet by touching the box at their preferred value point. Immediately after the selection, the box hiding the yellow token opened to reveal its location. This was accompanied by either the message ‘You win!’ and a short rising musical scale or ‘You lose!’ and a low tone. A winning choice was rewarded by the number of points bet, whereas a losing choice led to a loss of the points that had been bet. At the beginning of each block, the participant started off with a credit of 100 points and was instructed to win as many points as possible. Participants were informed that if scores reached 1 point on any one block, the block would terminate and the next would begin.

The gambling stage of the task included two modes of presentation of the bets: ascending first (i.e., the first bet that was offered was small and the subsequent bets offered were larger for two blocks, then in descending order for two blocks); and descending first (the first bet offered was large and subsequent bets offered were smaller for two blocks, then in ascending order for two blocks). Each bet represented a fixed percentage of the current total points score, but this was never made clear to the participant. On each trial, the participant was offered five bets whose order of presentation in the ascending condition was 5%, 25%, 50%, 75%, and 95% of the available points. In the descending condition, the order was reversed. In both conditions, each bet was presented along with a short tone with a pitch corresponding to the size of the bet; for example, a higher pitch indicating a higher bet. At the end of the sequence, if the participant had not selected a bet, the final bet in the sequence was placed automatically. *Risk Taking:* To maintain the independence of betting behavior and choice behavior, analyses were limited to the trials where the participants selected the color of the majority of boxes, i.e. trials on which they had more chance of winning than losing; *Risk Adjustment:* This was calculated as the degree to which the risk differed across the ratios, as a proportion of the overall amount risked by the participant: risk adjustment = [2*(% bet at 9:1) + (% bet at 8:2) – (% bet at 7:3) – 2*(% bet at 6:4)]/average % bet.

**Passive Avoidance Learning Task [20]***.* Participants had to learn by trial and error to press the key in response to the S + and to refrain from responding to the S –. The numbers were 2cm high, presented in white on a black screen. They stayed visible for 3 seconds if no response was made. Following Blair et al. [20], the sets of numbers were balanced; i.e., as many numbers even numbers were associated with reward as with punishment. If a response was made, a message appeared on the screen above the number, indicating how many points had been won or lost. All participants were allocated 10,000 points at the beginning of the task, and a running point total was visible on the screen during feedback only.

References

1. Wechsler D (1997) Weschsler Adult Intelligence Scale-III. San Antonio, TX: The Psychological Corporation.

2. Blair KS, Newman C, Mitchell DGV, Richell R, Leonard A, et al. (2006) Differentiating among prefrontal substrates in psychopathy: neuropsychological test findings. Neuropsychology 20: 153-165.

3. Barbey AK, Koenigs M, Grafman J (2012) Dorsolateral prefrontal contributions to human working memory. Cortex.

4. Séguin JR, Sylvers P, Lilienfeld S (2007) The neuropsychology of violence. In: Flannery DJ, Vazsonyi AT, Waldman ID, editors. The Cambridge handbook of violent behavior and aggression. New York: Cambridge University Press. pp. 187-214.

5. Giancola PR (1995) Evidence for dorsolateral and orbital prefrontal cortical involvement in the expression of aggressive behavior. Aggressive Behavior 21: 431-450.

6. Séguin JR, Pihl RO, Harden PW, Tremblay RE, Boulerice B (1995) Cognitive and neuropsychological characteristics of physically aggressive boys. Journal of Abnormal Psychology 104: 614-624.

7. Miller LA, Collins RL, Kent TA (2008) Language and the modulation of impulsive aggression. Journal of Neuropsychiatry and Clinical Neurosciences 20: 261-273.

8. Raine A, Moffitt TE, Caspi A, Loeber R, Stouthamer-Loeber M, et al. (2005) Neurocognitive impairments in boys on the life-course persistent antisocial path. Journal of Abnormal Psychology 114: 38-49.

9. Budhani S, Richell RA, Blair RJR (2006) Impaired reversal but intact acquisition: probabilistic response reversal deficits in adult individuals with psychopathy. Journal of Abnormal Psychology 115: 552-558.

10. Rolls ET (2000) The orbitofrontal cortex and reward. Cerebral Cortex 10: 284-294.

11. Blair RJR (2010) Psychopathy, frustration, and reactive aggression: The role of ventromedial prefrontal cortex. British Journal of Psychology 101: 383-399.

12. Rogers RD, Everitt BJ, Baldacchino A, Blackshaw AJ, Swainson R, et al. (1999) Dissociable deficits in the decision-making cognition of chronic amphetamine abusers, opiate abusers, patients with focal damage to prefrontal cortex, and tryptophan-depleted normal volunteers: Evidence for monoaminergic mechanisms. Neuropsychopharmacology 20: 322-339.

13. Clark L, Bechara A, Damasio H, Aitken MRF, Sahakian BJ, et al. (2008) Differential effects of insular and ventromedial prefrontal cortex lesions on risky decision-making. Brain 131: 1311-1322.

14. Mazas CA, Finn PR, Steinmetz JE (2000) Decision-making biases, antisocial personality, and early-onset alcoholism. Alcoholism: Clinical and Experimental Research 24: 1036-1040.

15. Mitchell DGV, Colledge E, Leonard A, Blair RJR (2002) Risky decisions and response reversal: Is there evidence of orbitofrontal cortex dysfunction in psychopathic individuals? Neuropsychologia 40: 2013-2022.

16. Dunn BD, Dalgleish T, Lawrence AD (2006) The somatic marker hypothesis: A critical evaluation. Neuroscience and Biobehavioral Reviews 30: 239-271.

17. Cervantes MC, Delville Y (2009) Serotonin 5-HT1A and 5-HT3 receptors in an impulsive-aggressive phenotype. Behavioral Neuroscience 123: 589-598.

18. Heatherton TF, Wagner DD (2011) Cognitive neuroscience of self-regulation failure. Trends in Cognitive Sciences 15: 132-139.

19. Solanto MV, Abikoff H, Sonuga-Barke E, Schachar R, Logan GD, et al. (2001) The ecological validity of delay aversion and response inhibition as measures of impulsivity in AD/HD: A supplement to the NIMH multimodal treatment study of AD/HD. Journal of Abnormal Child Psychology 29: 215-228.

20. Blair RJR, Mitchell DGV, Leonard A, Budhani S, Peschardt K, et al. (2004) Passive avoidance learning in individuals with psychopathy: Modulation by reward but not by punishment. Personality and Individual Differences 37: 1179-1192.

21. Ambrogi Lorenzini CG, Baldi E, Bucherelli C, Sacchetti B, Tassoni G (1999) Neural topography and chronology of memory consolidation: A review of functional inactivation findings. Neurobiology of Learning and Memory 71: 1-18.

22. Schoenbaum G, Roesch M (2005) Orbitofrontal Cortex, associative learning, and expectancies. Neuron 47: 633-636.

Footnote

^1^ While trials with a 5:5 ratio of red-blue boxes were included in the task in order to ensure that the participants perceived the task as a random trial sequence, these trials were excluded from statistical analyses.(K. S. Blair et al., 2006)
